# Association of Dietary Factors with Presence and Severity of Tinnitus in a Middle-Aged UK Population

**DOI:** 10.1371/journal.pone.0114711

**Published:** 2014-12-12

**Authors:** Abby McCormack, Mark Edmondson-Jones, Duane Mellor, Piers Dawes, Kevin J. Munro, David R. Moore, Heather Fortnum

**Affiliations:** 1 NIHR Nottingham Hearing Biomedical Research Unit, Ropewalk House, 113 The Ropewalk, Nottingham, United Kingdom; 2 Otology and Hearing group, Division of Clinical Neuroscience, School of Medicine, University of Nottingham, Nottingham, United Kingdom; 3 MRC Institute of Hearing Research, University Park, Nottingham, United Kingdom; 4 School of Biosciences, University of Nottingham, Nottingham, United Kingdom; 5 School of Psychological Sciences, University of Manchester, Manchester, United Kingdom; 6 Central Manchester University Hospitals NHS Foundation Trust, Manchester Academic Health Science Centre, Oxford Road, Manchester, United Kingdom; 7 Cincinnati Children's Hospital Medical Center, Cincinnati, Ohio, United States of America; University of Otago, New Zealand

## Abstract

**Objective:**

The impact of dietary factors on tinnitus has received limited research attention, despite being a considerable concern among people with tinnitus and clinicians. The objective was to examine the link between dietary factors and presence and severity of tinnitus.

**Design:**

This study used the UK Biobank resource, a large cross-sectional study of adults aged 40–69. 171,722 eligible participants were asked questions specific to tinnitus (defined as noises such as ringing or buzzing in the head or ears). Dietary factors included portions of fruit and vegetables per day, weekly fish consumption (oily and non-oily), bread type, cups of caffeinated coffee per day, and avoidance of dairy, eggs, wheat and sugar. We controlled for lifestyle, noise exposure, hearing, personality and comorbidity factors.

**Results:**

Persistent tinnitus, defined as present at least a lot of the time, was elevated with increased: (i) fruit/vegetable intake (OR = 1.01 per portion/day), (ii) bread (wholemeal/wholegrain, OR = 1.07; other bread, 1.20) and (iii) dairy avoidance (OR = 1.27). Persistent tinnitus was reduced with: (i) fish consumption (non-oily, OR = 0.91; oily, 0.95), (ii) egg avoidance (OR = 0.87) and (iii) caffeinated coffee consumption (OR = 0.99 per cup/day). Reports of “bothersome” tinnitus (moderate-severe handicap) increased with wholemeal/wholegrain bread intake (OR = 0.86). Reports of less frequent transient tinnitus increased with dairy avoidance (OR = 1.18) and decreased with caffeinated coffee (OR = 0.98 per cup/day) and brown bread (OR = 0.94).

**Conclusions:**

This is the first population study to report the association between dietary factors and tinnitus. Although individually dietary associations are mostly modest, particular changes in diet, such as switching between foodstuffs, may result in stronger associations. These findings offer insights into possible dietary associations with tinnitus, and this may be useful when discussing management options in combination with other lifestyle changes and therapies.

## Introduction

Tinnitus is the perception of sound without an external source. It affects 16% of middle-aged adults (40–69 years) in the UK [1], [2], and around 30% of adults aged over 75 years [3]. Tinnitus can be very distressing, and has been associated with anxiety and depression symptoms [4]–[6] and reduced quality of life [7]. Therefore, identifying modifiable risk factors for tinnitus is an important area of research that is of clinical relevance.

Research into the effect of diet on tinnitus was identified as a priority for future studies among both patients and clinicians [8]. A National Institute for Health Research (NIHR) research priority exercise, the James Lind Alliance (JLA), set up a partnership with the British Tinnitus Association, the Judi Meadows Memorial Fund, and the NIHR Nottingham Hearing Biomedical Research Unit. The aim was to determine what are the research priorities for tinnitus for both patients and clinicians [8], so that clinical research is relevant and beneficial to end users. According to the participants in the survey [8], the most common dietary questions were: ‘what is the link between tinnitus and diet?’; ‘what specific type of diet is effective in reducing tinnitus symptoms?’; ‘has cheese been proven to make tinnitus symptoms worse?’ and ‘is reduced caffeine intake proven to alleviate the symptoms of tinnitus?’ On the basis of that survey, dietary factors are a concern for tinnitus patients.

There is some limited literature exploring associations between dietary factors and hearing in humans [9]–[15], which suggests that diets high in unsaturated fats may reduce the risk of hearing loss, as do lower Glycaemic Index (GI) diets, i.e. wholegrain foods, fruits and vegetables. However, we are not aware of any studies exploring this association with tinnitus. As tinnitus is associated with hearing loss [16], it is possible that the same dietary factors implicated with hearing loss would also be important for tinnitus.

There is no empirical research, nor any hypothesised mechanism, to link food or (non-alcoholic) drinks to the presence or severity of tinnitus. However, many tinnitus patients believe certain foods or drinks can trigger or exacerbate their tinnitus symptoms, while others believe the same foods may reduce their tinnitus [17]. One example is coffee; some tinnitus patients suspect that coffee consumption may exacerbate tinnitus. However, scientific evidence of a link between coffee consumption and tinnitus is limited, and no possible mechanism has been suggested. Caffeine abstinence as a possible treatment for tinnitus has been explored [18]. In that study, 66 tinnitus participants who usually consumed at least 150 mg/day of caffeine participated in a pseudo-randomized, double-blinded, placebo-controlled crossover trial over 30 days [18]. Their usual caffeinated drink was replaced with double-blinded supplies under one of two conditions: maintenance followed by phased withdrawal, or phased withdrawal followed by reintroduction and maintenance. The total score on the Tinnitus Questionnaire [19] on days 1, 15, and 30, was used as the outcome measure of tinnitus severity. Caffeine had no effect on tinnitus severity. No evidence was found to justify caffeine abstinence as a therapy to alleviate tinnitus. Furthermore, a recent review of the role of caffeine in otorhinolaryngological conditions found no evidence to support the notion that caffeine causes or aggravates tinnitus [20]. Recent evidence suggests that caffeine may actually have a protective effect against tinnitus. A large prospective study with 65,085 females, found that those who drank more caffeine had lower odds of having tinnitus [21]. There are no other studies examining the association between tinnitus and other dietary factors, e.g. fruit and vegetables, breads and fish.

The present study explores potential associations between food-based dietary factors and tinnitus, in a large representative cross-sectional cohort of UK adults aged 40 to 69 years. Based on the limited research, we predict that caffeinated coffee consumption will be associated with reduced odds of current and bothersome tinnitus. Although no hypothesised mechanisms for other associations have been suggested we also explore the associations between current and bothersome tinnitus with consumption of different types of bread, fruit and vegetables, fish, and avoidance of dairy, eggs, wheat and sugar. The study identifies patterns of diet that are associated with increased risk of tinnitus, and this may offer options for advice or self-management. Population evidence of associations between diet and tinnitus may offer avenues for prevention or remediation.

## Method

This research was covered by the UK Biobank ethics agreement (http://www.ukbiobank.ac.uk/). Within England, UK Biobank has approval from the North West Multi-centre Committee (MREC). All participants provided written informed consent.

### Study population

More than 500,000 people aged 40–69 years were recruited to the UK Biobank cohort study during 2006–2010. The aim of UK Biobank was to provide a health data resource to improve the prevention, diagnosis and treatment of a wide range of serious and life threatening illnesses [22]. Data were collected on age, sex and Townsend deprivation scores based on area of residence as a proxy measure of socioeconomic status. (Townsend deprivation score is a geography based measure providing a measure of deprivation based on the 2001 census returns.) As part of the assessment, participants completed a touchscreen questionnaire that included questions on diet and tinnitus.

### Tinnitus assessment

Two self-report tinnitus questions were introduced part way through data collection, and were presented to 171,722 eligible participants. The first question was: ‘Do you get or have you had noises (such as ringing or buzzing) in your head or in one or both ears that lasts for more than five minutes at a time?’ Those who answered yes were then asked to categorise the duration from a predefined set of responses. Persistent tinnitus was identified if the participant responded: ‘Yes, now most or all of the time’ or ‘Yes, now a lot of the time’ and transient tinnitus was defined in relation to those who responded ‘Yes, now some of the time’. The second question was concerned with severity of the tinnitus: ‘How much do these noises worry, annoy or upset you when they are at their worst?’ Bothersome tinnitus was defined as those who responded ‘severely’ or ‘moderately’. The other options were ‘slightly’ and ‘not at all’. The prevalence of bothersome tinnitus was investigated only amongst those reporting persistent tinnitus.

### Dietary data

The development of the dietary questions within the UK Biobank touchscreen questionnaire involved selecting a relatively short set of food frequency questions to rank participants according to commonly eaten food groups. This was based on the expected distribution in the British population, derived from the National Diet and Nutrition Survey [23]. This approach also seeks information about some common sources of various nutrients, but recognises that it does not allow assessment of total energy intake or some specific nutrients. This non-validated questionnaire was assessed for construct validity as part of the UK Biobank pilot evaluation (UK Biobank Co-ordinating Centre, 2006). Questions were semi-quantitative (e.g. number of tablespoons) and asked for intakes of fruit, vegetables, fish (including oily fish), bread, avoidance of dairy, eggs, wheat and sugar, and coffee consumption. See S1 Table for full list of online dietary questions.

#### Fruit and vegetables

Based on the UK guidelines [24] that a portion of vegetables is three heaped tablespoons, the data were recoded to calculate how many portions of cooked vegetables, salad and raw vegetables were consumed in an average day. Each piece of fruit counted as one portion [24]. Fruit and vegetables were analysed as a continuous variable as the number of portions eaten per day.

#### Fish

A variable was created for non-oily fish (and a separate variable for oily fish) consumption with two categories each: less than once a week; and once a week or more.

#### Bread

Bread was analysed as a categorical variable with four levels: white; brown; wholemeal or wholegrain; and other type.

#### Dairy, eggs, wheat and sugar

Four new dichotomous variables were created for ‘dairy avoidance’; ‘egg avoidance’; ‘wheat avoidance’; and ‘sugar avoidance’, with the categories ‘yes’ and ‘no’ according to whether respondents reporting never consuming each.

#### Coffee

We created a new variable of number of cups of caffeinated coffee per day. Those who indicated drinking decaffeinated coffee were put in the zero cups group.

### Potential confounds

Potential confounds that were controlled for included smoking status, hearing ability, occupational and leisure noise exposure, medical conditions, body mass index (BMI), body fat percentage and the personality trait of neuroticism.

#### Smoking status

Participants were asked about their smoking habits. Those who responded to the question ‘Do you smoke tobacco now?’ by answering ‘Yes, on most or all days’ or ‘Only occasionally’ were classified as current smokers. Otherwise those who responded to the question ‘In the past, how often have you smoked tobacco?’ by answering ‘Smoked on all or most days’ or ‘Smoked occasionally’ were classified as past smokers, while those who answered ‘Just tried once or twice’ or ‘I have never smoked’ were classified as never smokers.

#### Hearing loss

Approximately 96% of the participants (n = 165,604) completed a test of speech in noise (SiN) hearing, a truncated digit triplet test (DTT) with fifteen triplets of monosyllabic digits (e.g. 2-9-6) presented to each ear separately using circumaural headphones (Sennheiser D25) against a background of noise spectrally matched to the corpus of digits with the signal-to-noise ratio (SNR) adaptively varied [25]. Performance was measured in terms of the mean SNR from the last eight triplets presented to each ear. Hearing was classified as normal if the better ear speech recognition threshold (SRT) was lower than two standard deviations above that of a reference group of participants aged 18–29 years with normal hearing (defined as pure tone audiometric thresholds <25 dB HL between 250 Hz and 8,000 Hz bilaterally), poor if the better ear SRT was more than 2 dB above this reference level, and insufficient otherwise [2].

#### Noise exposure

Occupational noise exposure and music-related noise exposure were identified on the basis of any reported noise exposure in response to the questions ‘Have you ever worked in a noisy place where you had to shout to be heard?’ and ‘Have you ever listened to music for more than 3 hours per week at a volume which you would need to shout to be heard or, if wearing headphones, someone else would need to shout for you to hear them?’

#### Medical conditions, BMI and body fat percentage

Comorbidities were recorded within the online self-completed questionnaire. Diabetes was identified on the basis of self-report of either Type 1 or Type 2 diabetes (gestational diabetes was excluded from the analysis), or if people reported currently taking insulin for diabetes. Cardiovascular disease was identified on the basis of self-report of the following cardiovascular problems: angina, heart attack, heart failure, stroke, transient ischaemic attack, leg claudication or intermittent claudication, arterial embolism, pulmonary embolism, venous thrombotic disease, or deep venous thrombosis. High cholesterol was identified if the participant reported that they had high cholesterol, or that they were currently taking medication to treat it. Hypertension was identified if the participant reported that they had hypertension, had a measured systolic blood pressure greater than 140 mm Hg or diastolic pressure greater than 90 mm Hg (mean over two readings; manual or automated), or reported currently taking medication for hypertension or to lower blood pressure. Body mass index (BMI) was calculated as the participants weight (in kilograms) divided by height (in metres) squared. Body fat percentage was taken by impedance measurement.

#### Neuroticism symptoms

Previous research has shown a significant association between the persistent personality trait of neuroticism and experience of tinnitus symptoms within the UK Biobank study [1]. Participants were asked a number of questions relating to the neuroticism dimension of the Eysenck Personality Questionnaire. These comprised thirteen questions relating to mood swings, miserableness, irritability, sensitivity/hurt feelings, fed-up feelings, nervous feelings, worrying/anxious feelings, tense/‘highly strung’, worrying too long after embarrassment, suffering from ‘nerves’, loneliness/isolation, guilty feelings and risk taking. The number of ‘Yes’ responses was summed and a classification allocated where 0–2 corresponded to ‘Low’, 3–5 to ‘Low-medium’, 6–9 to ‘Medium-high’ and 10–13 to ‘High’.

### Statistical analysis

The association between presence of each of transient tinnitus, persistent tinnitus and bothersome tinnitus, and dietary intakes of fruit and vegetables, breads, fish, coffee, and avoidance of dairy, wheat, eggs, and sugar, were examined in three separate logistic regression models while controlling for the potential confounders: smoking status, age (5-year age bands), sex, Townsend deprivation score (national quintiles), hearing difficulty (measured by impaired SRT), noise exposure, BMI, body fat percentage, diabetes, cardiovascular disease, hypertension, high cholesterol and neuroticism symptoms. For each of transient tinnitus and persistent tinnitus the comparison is made only against those not reporting current tinnitus. For bothersome tinnitus only those reporting persistent tinnitus were assessed. In order to avoid bias, allowing for data not being missing completely at random, missing responses were modelled using multiple imputation with 25 imputed complete datasets [26]. Analyses were performed using R 3.1.0 and the package mice. SPSS 21 was used to pre-process the data. Results of logistic regression analysis are expressed as odds ratios (ORs) with 95% CIs. Intake was analysed by categorizing participants according to the frequency of consumption of servings. Statistical significance was defined as *p*<0.05.

## Results

Table 1 shows the characteristics of the total sample according to their dietary intake in participants with and without tinnitus.

**Table 1:** Epidemiologic, dietary, and clinical characteristics of participants.

		Tinnitus			
	N	None	Transient	Constant	Missing
**Dietary factors**	171,722	137,825	14,921	14,714	4,262
Fruit and vegetable (portions/day)	166,573	4.70 (sd = 3.18)	4.73 (sd = 3.31)	4.80 (sd = 3.44)	4.80 (sd = 3.87)
Non-oily fish	170,585	65.5%	65.7%	64.4%	61.1%
Oily fish	170,521	55.9%	56.3%	55.8%	49.6%
Type of bread	165,171				
- White		26.0%	28.3%	27.3%	33.5%
- Brown		12.7%	12.6%	12.1%	17.3%
- Wholemeal/wholegrain		56.9%	54.3%	55.8%	42.9%
- Other		4.4%	4.9%	4.8%	6.3%
Avoidance					
- Dairy	170,959	2.2%	2.9%	3.0%	3.8%
- Eggs	170,959	2.8%	3.2%	3.0%	5.8%
- Wheat	170,959	2.6%	3.2%	3.1%	3.5%
- Sugar	170,959	17.4%	18.9%	19.6%	16.4%
Caffeinated coffee (cups/day)	170,813	1.59	1.54	1.56	1.43
		2.03	2.06	2.03	2.05
**Potential confounds**					
Age (yrs)	171,722	56.3 (sd = 8.17)	57.8 (sd = 7.80)	59.3 (sd = 7.20)	55.2 (sd = 8.49)
Sex (Males)	171,722	43.9%	47.9%	57.7%	48.4%
Townsend index	171,439	−1.15 (sd = 2.93)	−0.88 (sd = 3.07)	−1.12 (sd = 2.96)	0.16 (sd = 3.34)
Occupational noise exposure	169,491	20.5%	32.5%	36.4%	30.8%
Loud music exposure	168,644	11.5%	16.9%	16.5%	14.9%
Speech-in-Noise hearing	164,756				
- Normal		88.4%	83.5%	80.0%	81.2%
- Insufficient		10.1%	13.4%	15.7%	15.3%
- Poor		1.5%	3.1%	4.3%	3.6%
Smoking status	170,963				
- Current		10.1%	11.0%	9.4%	13.7%
- Past		33.8%	36.5%	40.1%	29.8%
- Never		56.1%	52.5%	50.5%	56.5%
Body Mass Index (BMI)	167,550	27.4 (sd = 4.80)	27.7 (sd = 4.95)	27.7 (sd = 4.68)	28.0 (sd = 5.07)
Body fat percentage	167,458	31.7 (sd = 8.54%)	31.8 (sd = 8.75%)	30.7 (sd = 8.52%)	32.0 (sd = 8.68%)
Cardiovascular problems	171,722	7.7%	10.8%	12.3%	10.4%
Hypertension	171,722	56.4%	61.4%	64.3%	58.2%
Diabetes	171,000	5.3%	6.5%	6.7%	9.9%
High cholesterol	171,722	18.7%	23.5%	26.5%	22.2%
Neuroticism symptoms	135,326	4.27 (sd = 3.24)	5.01 (sd = 3.46)	4.85 (sd = 3.38)	5.21 (sd = 3.62)

Table 2 shows results from the logistic regression models with transient, persistent and bothersome tinnitus as the dependent variable. The models were controlled for age, sex, Townsend deprivation, noise exposure, hearing difficulty, smoking status, BMI, body fat percentage, diabetes, cardiovascular disease, hypertension and high cholesterol, and neuroticism symptoms. Each of transient and persistent tinnitus are compared against those reporting no current tinnitus. Bothersome tinnitus relates only to those reporting persistent tinnitus. Odds ratios and 95% confidence intervals are shown.

**Table 2:** Logistic regression of dietary factors predicting likelihood of tinnitus.

	Transient tinnitus		Persistent tinnitus		Bothersome tinnitus	
Food Type	Odds ratio	p-value	Odds ratio	p-value	Odds ratio	p-value
Fruit and vegetable (portions/day)	1.00 (1.00–1.01)	0.759	**1.01 (1.00–1.01)**	**0.018**	1.01 (1.00–1.02)	0.073
Non-oily fish once or more/week	0.98 (0.95–1.02)	0.385	**0.91 (0.87–0.94)**	**<0.001**	1.08 (0.99–1.16)	0.071
Oily fish once or more/week	1.00 (0.97–1.04)	0.813	**0.95 (0.91–0.99)**	**0.010**	0.95 (0.88–1.03)	0.195
Bread type (reference: White)						
- Brown	**0.94 (0.88–1.00)**	**0.034**	0.95 (0.89–1.01)	0.108	1.05 (0.93–1.19)	0.430
- Wholemeal/wholegrain	0.96 (0.92–1.01)	0.098	**1.07 (1.02–1.12)**	**0.004**	**0.86 (0.79–0.94)**	**0.001**
- Other	1.07 (0.98–1.17)	0.115	**1.20 (1.10–1.31)**	**<0.001**	0.99 (0.83–1.18)	0.897
Food avoidance						
- Dairy	**1.18 (1.05–1.33)**	**0.004**	**1.27 (1.13–1.42)**	**<0.001**	1.14 (0.91–1.43)	0.259
- Eggs	0.97 (0.87–1.08)	0.621	**0.87 (0.77–0.97)**	**0.011**	1.11 (0.88–1.39)	0.385
- Wheat	1.06 (0.95–1.18)	0.275	1.03 (0.93–1.16)	0.547	1.23 (0.99–1.53)	0.059
- Sugar	1.00 (0.96–1.05)	0.952	0.99 (0.94–1.03)	0.547	1.03 (0.94–1.13)	0.532
Caffeined coffee (cups/day)	**0.98 (0.97–0.99)**	**<0.001**	**0.99 (0.98–0.99)**	**0.001**	0.99 (0.97–1.01)	0.198
**Potential confounds**						
Age group (reference: 40–44)						
- 45–49	**1.12 (1.04–1.22)**	**0.005**	**1.39 (1.26–1.53)**	**<0.001**	0.84 (0.68–1.03)	0.090
- 50–54	**1.31 (1.21–1.41)**	**<0.001**	**1.87 (1.70–2.05)**	**<0.001**	0.97 (0.80–1.17)	0.751
- 55–59	**1.51 (1.40–1.62)**	**<0.001**	**2.44 (2.24–2.67)**	**<0.001**	0.92 (0.76–1.11)	0.363
- 60–64	**1.68 (1.56–1.81)**	**<0.001**	**2.95 (2.70–3.22)**	**<0.001**	0.86 (0.72–1.03)	0.106
- 65–69	**1.80 (1.67–1.95)**	**<0.001**	**3.41 (3.11–3.73)**	**<0.001**	**0.80 (0.66–0.96)**	**0.018**
Sex (Male)	1.05 (0.98–1.13)	0.176	**1.60 (1.49–1.73)**	**<0.001**	**0.86 (0.73–1.00)**	**0.046**
Deprivation quintile (reference: 1 = least)						
- 2	1.05 (1.00–1.10)	0.050	0.99 (0.95–1.04)	0.682	1.07 (0.97–1.17)	0.175
- 3	1.02 (0.97–1.07)	0.515	0.97 (0.92–1.02)	0.215	1.00 (0.90–1.11)	0.982
- 4	**1.12 (1.06–1.18)**	**<0.001**	1.00 (0.95–1.06)	0.986	**1.16 (1.05–1.29)**	**0.005**
- 5 = most	**1.21 (1.13–1.29)**	**<0.001**	0.98 (0.91–1.05)	0.534	**1.24 (1.08–1.42)**	**0.002**
Occupational noise exposure	**1.65 (1.58–1.72)**	**<0.001**	**1.75 (1.68–1.82)**	**<0.001**	**1.43 (1.32–1.55)**	**<0.001**
Loud music exposure	**1.51 (1.43–1.59)**	**<0.001**	**1.56 (1.48–1.64)**	**<0.001**	1.07 (0.97–1.18)	0.179
Speech-in-Noise hearing (reference: Normal)						
- Insufficient	**1.23 (1.17–1.30)**	**<0.001**	**1.45 (1.37–1.52)**	**<0.001**	**1.27 (1.15–1.41)**	**<0.001**
- Poor	**1.67 (1.50–1.86)**	**<0.001**	**2.30 (2.08–2.54)**	**<0.001**	**1.74 (1.46–2.07)**	**<0.001**
Smoking status (reference: Current)						
- Past	1.01 (0.95–1.07)	0.861	**1.18 (1.11–1.26)**	**<0.001**	0.91 (0.80–1.03)	0.132
- Never	1.00 (0.94–1.06)	0.874	**1.12 (1.05–1.20)**	**<0.001**	**0.86 (0.75–0.97)**	**0.016**
Body Mass Index (BMI)	1.00 (0.99–1.01)	0.777	**0.99 (0.98–1.00)**	**0.004**	1.00 (0.98–1.01)	0.504
Body fat percentage	1.01 (1.00–1.01)	0.050	**1.01 (1.00–1.01)**	**0.003**	**1.02 (1.01–1.03)**	**<0.001**
Cardiovascular problems	**1.15 (1.08–1.22)**	**<0.001**	**1.18 (1.11–1.25)**	**<0.001**	**1.34 (1.20–1.50)**	**<0.001**
Hypertension	**1.04 (1.00–1.08)**	**0.045**	1.01 (0.98–1.06)	0.468	**0.87 (0.80–0.94)**	**<0.001**
Diabetes	0.98 (0.90–1.06)	0.587	**0.90 (0.84–0.98)**	**0.013**	1.01 (0.87–1.19)	0.866
High cholesterol	**1.05 (1.00–1.11)**	**0.034**	**1.07 (1.02–1.12)**	**0.004**	**1.15 (1.04–1.26)**	**0.004**
Neuroticism symptoms (reference: Low)						
- Low-medium	**1.16 (1.10–1.22)**	**<0.001**	**1.26 (1.20–1.33)**	**<0.001**	**1.45 (1.29–1.62)**	**<0.001**
- Medium-high	**1.37 (1.30–1.45)**	**<0.001**	**1.46 (1.37–1.56)**	**<0.001**	**1.91 (1.69–2.17)**	**<0.001**
- High	**1.67 (1.55–1.81)**	**<0.001**	**1.71 (1.56–1.87)**	**<0.001**	**2.56 (2.17–3.02)**	**<0.001**

As expected, all potential confounding variables were associated with at least one outcome. These results have been reported elsewhere [1], [2]. A number of results are of particular note. Past and never-smokers were at a significantly increased risk of persistent tinnitus, but not transient tinnitus, although the additional association of never-smokers appeared to be largely with regard to non-bothersome tinnitus. Body fat percentage was associated with increased reporting of both persistent and bothersome tinnitus, but not transient tinnitus, whereas BMI was associated with a reduction in the prevalence of persistent tinnitus. However, these odds ratios are relatively small per % body fat and unit of BMI, and would have minimal effect in relation to typical participants. Prevalence of transient, persistent and bothersome tinnitus all increased markedly in line with increasing numbers of neuroticism symptoms, and to a lesser extent in the presence of reported cardiovascular problems or high cholesterol. Conversely, indications of hypertension were associated with a small increase in the prevalence of transient, but not persistent, tinnitus however there was a reduction in the prevalence of bothersome tinnitus. Reported diabetes was associated with a reduced risk of persistent tinnitus, but not with transient or bothersome tinnitus.

### Transient tinnitus

Avoidance of dairy produce was found to be significantly associated with experience of transient tinnitus, whereas consumption of caffeinated coffee appeared to be associated with lower levels of reported transient tinnitus. Although the odds ratio for caffeinated coffee consumption was relatively small at 0.98/cup this effect is compounded by the number off cups consumed. (A fifth of respondents reported consuming 3 or more cups per day.) Brown bread consumption was found to have a marginal association with transient tinnitus reporting, relative to consumption of white bread, but wholemeal/wholegrain and other bread types did not. No association was seen between transient tinnitus and increased consumption of fruit and vegetables or fish (oil or non-oily) or with avoidance of eggs, wheat and sugar.

### Persistent tinnitus

Eating oily fish, or non-oily fish once a week or more, avoiding eggs, and drinking more cups of caffeinated coffee per day were associated with a lower odds ratio of reporting persistent tinnitus. Conversely greater consumption of fruit and vegetables per day, eating wholemeal/wholegrain or ‘other’ type of bread compared to white bread, and avoidance of dairy produce were associated with increased report of persistent tinnitus. No evidence was found that avoidance of wheat and sugar, or eating brown bread was associated with experiencing persistent tinnitus. Odds ratios were relatively small for both fruit and vegetable consumption and for consumption of caffeinated coffee even when compounded across the range of typical consumption levels. For example, fruit and vegetable consumption of five portions per day would be associated with a modelled odds ratio of 1.03, and caffeinated coffee consumption of three cups per day would be associated with a modelled odds ratio of 0.96.

### Bothersome tinnitus

The only dietary association with reporting of bothersome persistent tinnitus was a reduction in prevalence when wholemeal/wholegrain bread was consumed rather than white or brown bread. Furthermore, the reduction in odds of experiencing bothersome tinnitus is similar in magnitude to the increase in odds of experiencing persistent tinnitus, suggesting that any associated increase in reporting of persistent tinnitus is largely non-bothersome in nature.

## Discussion

Although dietary associations are relatively modest this is, to our knowledge, the first population study to explore associations between different food types and tinnitus using a large representative cohort of adults aged 40 to 69 years. Based on the limited literature exploring associations with dietary factors, and anecdotal suspicions from clinicians and patients [8], we hypothesised that caffeinated coffee consumption would be associated with reduced odds of current and bothersome tinnitus. We also aimed to explore the associations between current and bothersome tinnitus with consumption of different types of bread, fruit and vegetables, fish, and avoidance of dairy, eggs, wheat and sugar.

Our study did find that caffeinated coffee was associated with a lower prevalence of tinnitus, both transient and persistent, although the odds ratio was relatively small across the range of typical consumption. Those who drink more cups of coffee per day were less likely to report tinnitus. This supports findings from a recent prospective study [21] which found that females who drank more coffee had lower odds of tinnitus. It is more likely that withdrawal of caffeine could potentially worsen symptoms of tinnitus [18]. Further randomised controlled trials are needed to help explain the mixed findings, and to explore links with lifestyle factors.

We report various diverse associations between possible risk factors and each of transient, persistent and bothersome tinnitus. Eating oily fish, and/or non-oily fish once a week or more was associated with a reduction in the odds of persistent tinnitus but we could demonstrate no association with either transient or bothersome tinnitus. Eating a greater number of portions of fruit and vegetables per day was not associated with transient or bothersome tinnitus, but was slightly associated with increased odds of persistent tinnitus. Compared to those who ate white bread, those who ate brown bread had reduced odds of transient tinnitus whereas those who ate wholemeal/wholegrain or ‘other bread’ had increased odds of persistent tinnitus. Only consumption of wholemeal/wholegrain bread had a differential association with bothersome tinnitus when compared to white bread consumption. It is recommended that wholegrain and wholemeal varieties are chosen, as these have lower GI scores [24]. However it is possible that participants were not familiar with the differences between brown bread and wholegrain/wholemeal bread and may have indicated eating brown bread when in fact they were eating wholemeal bread, as there is evidence that people do not have a good general knowledge of nutrition and food intake [27]. Associations between reports of tinnitus and wholemeal or wholegrain bread consumption are complex; consumption is associated with increased persistent tinnitus, but reduced report of bothersomeness among these cases, consistent with any increase in persistent tinnitus being predominantly reported as non-bothersome. We note however that the UK Biobank method is not sensitive enough to distinguish amounts of glycaemic load. The initial dataset of UK Biobank had limited information on nutritional intake including no data on other starchy foods, e.g. rice, potatoes or pasta. Research has shown that a high GI diet has been associated with an increased prevalence of hearing loss [10]. Prospective data collected using a comprehensive food frequency questionnaire will allow for associations with tinnitus to be assessed within the UK Biobank sample.

Avoidance of dairy produce was associated individually with increased odds of transient and persistent tinnitus, but not with bothersome tinnitus. There are two ways of interpreting this; avoiding dairy increases the risk of tinnitus, or those with tinnitus avoid dairy in an attempt to reduce their symptoms. Further prospective work is needed. There was a moderate association between avoidance of eggs and reduced reporting of persistent tinnitus, but not with either transient or bothersome tinnitus. This association could, hypothetically, be linked to eggs as a source of dietary cholesterol, and may be consistent with individuals reporting high cholesterol being more likely to report tinnitus symptoms. There was no association with avoidance of wheat or sugar, and transient or persistent tinnitus. It is possible that where certain foods are avoided substitute food types (e.g. ‘other’ bread types, dairy or fruit in place of wheat or sugar) might be more directly associated with tinnitus reporting than the absence of what they are replacing. From Table 1 we do see that those reporting tinnitus symptoms are more likely to avoid certain food types. However in the case of wheat or sugar we see no evidence suggesting that this is associated with any significant improvement. It is possible that the severity of the tinnitus may be associated with mental health such as depression and anxiety symptoms [28], perhaps as a consequence of personality factors [1]. It is possible that those who are predisposed to be more bothered by their tinnitus make more of a conscious effort to avoid certain things in their diet in an attempt to control their symptoms and that experience of tinnitus is a complex interaction between personality and related factors such as diet.

### Strengths and limitations

The key strength of the present study is the large and representative sample. This is the first study to examine the relationship between aspects of diet and tinnitus. Our study also examines intakes of food items as consumed rather than nutrients, and is one of the first studies to report such a comprehensive analysis in humans. A focus on nutrients could be a limitation and it has been argued that focusing on real foods may actually be more beneficial [15] because they are more relevant and controllable for individuals. A focus on real foods is a more common approach within nutritional epidemiological studies, and may be more representative of population food intake.

However, as a cross-sectional study, this work can only suggest associations of diet with tinnitus reporting and generate hypotheses that may be tested using controlled experimental designs. It is difficult in observational studies, to separate the effects of single food items on the development of disease, because foods are consumed in combination, resulting in different net effects. Furthermore, this study only considered caffeine intake through coffee, and not other sources such as tea, chocolate, or soda drinks. The caffeinated coffee drinkers may also have consumed caffeine through other means. The food questionnaire that was used was not validated with other dietary methods, e.g. food diaries or biomarkers. It is highly probable that diet is associated with a range of lifestyle factors that were not measured, therefore associations could be confounded with factors that were not fully accounted for. Total calorie intake was also not assessed. A potential limitation is the fact that no information was available on the duration that the participant experienced tinnitus, their age at onset, or information on lateralization, pitch, and loudness of the tinnitus. It is therefore possible that grouping all tinnitus patients together hides any associations that may be present.

Our results show relatively small associations between diet and tinnitus in comparison with strength of associations between tinnitus and age, hearing, noise exposure and personality. These findings might provide some insight into associations but are not sufficient to indicate potential mechanisms. What they do offer is some background data for clinicians who are discussing management options with patients who have tinnitus to enable them to offer some management strategies to such patients. This study suggests that carefully monitoring their diet may help individual tinnitus patients identify foods that aggravate or reduce their tinnitus symptoms. However, as yet there are no firm recommendations to suggest to all tinnitus patients that they add or remove certain food groups from their diet. Our next step is to conduct further analysis on dietary factors derived from the food frequency questionnaire introduced as a follow up of a subset of participants in the UK Biobank study [22].

### Conclusions

The impact of dietary factors on tinnitus has received little empirical attention. Our study is the first population study to examine the association between dietary factors and tinnitus. The results of this study, taken in context with the limited existing literature, suggest that there may be a benefit of modifying diet, albeit small due to the size of association. However, due to the limitations mentioned, it still remains to be investigated whether dietary factors could lead to improvements in tinnitus. The results of this study suggest areas for investigation but cannot definitively confirm whether some foods should be avoided to reduce tinnitus symptoms. However clinicians may want to advise tinnitus patients to carefully monitor their diet to note whether any food aggravates or reduces their tinnitus. Further, high quality randomised controlled trials could determine whether tinnitus benefits are derived from dietary factors, such as fish consumption, glycaemic load, dairy avoidance and caffeine intake. Associations between food intake and hearing problems do warrant further study in prospective cohort studies and longitudinal studies, to potentially identify modifiable factors that could prevent or delay tinnitus in adults.

## Supporting Information

S1 Table **Dietary data in the UK Biobank study.**
(DOCX)
