# Correction: Association of Dietary Factors with Presence and Severity of Tinnitus in a Middle-Aged UK Population

**DOI:** 10.1371/journal.pone.0123053

**Published:** 2015-04-01

**Authors:** The PLOS ONE Staff

There is an error in the penultimate sentence of the Results section of the Abstract. The correct sentence is: Reports of “bothersome” tinnitus (moderate-severe handicap) reduced with wholemeal/wholegrain bread intake (OR = 0.86).
