# Supplementary material for: Association of Dietary Factors with Presence and Severity of Tinnitus in a Middle-Aged UK Population
Source: PLoS One. 2014 Dec 12;9(12):e114711. doi: 10.1371/journal.pone.0114711 (PMC4264778; doi:10.1371/journal.pone.0114711)
Supplement: S1 Table — Dietary data in the UK Biobank study. (DOCX) [file pone.0114711.s001.docx]

**Table S1**

| **Food type** | **Question** | **Response** | **Comments** |
| --- | --- | --- | --- |
| Cooked vegetable intake | On average how many heaped tablespoons of COOKED vegetables would you eat per DAY? | Open | Not including potatoes |
| Salad/raw vegetable intake | On average how many heaped tablespoons of SALAD or RAW vegetables would you eat per DAY? | Open | Include lettuce and tomato in sandwiches |
| Dried fruit intake | About how many pieces of DRIED fruit would you eat per DAY? | Open | 1 prune, 1 dried apricot or 10 raisins etc = 1 piece |
| Fresh fruit intake | About how many pieces of FRESH fruit would you eat per DAY? | Open | 1 apple, 1 banana, 10 grapes etc =- 1 piece |
| Oily fish intake | How often do you eat oily fish? (e.g. sardines, salmon, mackerel, herring) | Never/Less than once a week/Once a week/2-4 times a week/5-6 times a week/Once or more daily |  |
| Non oily fish intake | How often do you eat other types of fish? (e.g. cod, tinned tuna, haddock) | Never/Less than once a week/Once a week/2-4 times a week/5-6 times a week/Once or more daily |  |
| Food avoidance | Which of the following do you NEVER eat? | Eggs or foods containing eggs/Dairy products/Wheat products/Sugar or foods/drinks containing sugar/I eat all of the above |  |
| Bread intake | How many slices of bread do you eat each WEEK? | Open |  |
| Bread type | What type of bread do you mainly eat? | White/Brown/Wholemeal or wholegrain/Other type of bread |  |
| Coffee intake | How many cups of coffee do you drink each DAY? | Open |  |
| Coffee type | What type of coffee do you usually drink? | Decaffeinated coffee (any type)/Instant coffee/Ground coffee (include espresso, filter etc) /Other type of coffee |  |
